# Juvenile Plant–Microbe Interactions Modulate the Adaptation and Response of Forest Seedlings to Rapid Climate Change

**DOI:** 10.3390/plants13020175

**Published:** 2024-01-09

**Authors:** Tedy Sanhueza, Ionel Hernández, Cristiane Sagredo-Sáez, Angela Villanueva-Guerrero, Roxana Alvarado, Maria Isabel Mujica, Alejandra Fuentes-Quiroz, Esther Menendez, Emilio Jorquera-Fontena, Rafael Borges da Silva Valadares, Héctor Herrera

**Affiliations:** 1 Laboratorio de Silvicultura, Departamento de Ciencias Forestales, Facultad de Ciencias Agropecuarias y Medioambiente, Universidad de La Frontera, Temuco 4811230, Chile; tedy.sanhueza@ufrontera.cl (T.S.); c.sagredo03@ufromail.cl (C.S.-S.); angela.villanueva@ufrontera.cl (A.V.-G.); roxanalvaradoab@gmail.com (R.A.); alejandra.fuentes@ufrontera.cl (A.F.-Q.); 2 Plant Physiology and Biochemistry Department, National Institute of Agricultural Science, Carretera a Tapaste Km 3 y ½, San José de las Lajas 32700, Mayabeque, Cuba; ionel.hdez09@gmail.com; 3 Instituto de Ciencias Ambientales y Evolutivas, Universidad Austral de Chile, Valdivia 5110566, Chile; mujisa@gmail.com; 4 Departamento de Microbiología y Genética, Instituto de Investigación en Agrobiotecnología (CIALE), Universidad de Salamanca, 37008 Salamanca, Spain; esthermenendez@usal.es; 5 Departamento de Ciencias Agropecuarias y Acuícolas, Facultad de Recursos Naturales, Universidad Catolica de Temuco, Temuco P.O. Box 15-D, Chile; ejorquera@uct.cl; 6 Instituto Tecnologico Vale, Rua Boaventura da Silva 955, Belém 66050-090, PA, Brazil; rafael.borges.valadares@itv.org; 7 Laboratorio de Ecosistemas y Bosques, Departamento de Ciencias Forestales, Facultad de Ciencias Agropecuarias y Medioambiente, Universidad de La Frontera, Temuco 4811230, Chile

**Keywords:** bacteria, endophytes, fungi, reforestation, rhizosphere, seed germination, symbiosis

## Abstract

The negative impacts of climate change on native forest ecosystems have created challenging conditions for the sustainability of natural forest regeneration. These challenges arise primarily from abiotic stresses that affect the early stages of forest tree development. While there is extensive evidence on the diversity of juvenile microbial symbioses in agricultural and fruit crops, there is a notable lack of reports on native forest plants. This review aims to summarize the critical studies conducted on the diversity of juvenile plant–microbe interactions in forest plants and to highlight the main benefits of beneficial microorganisms in overcoming environmental stresses such as drought, high and low temperatures, metal(loid) toxicity, nutrient deficiency, and salinity. The reviewed studies have consistently demonstrated the positive effects of juvenile plant–microbiota interactions and have highlighted the potential beneficial attributes to improve plantlet development. In addition, this review discusses the beneficial attributes of managing juvenile plant–microbiota symbiosis in the context of native forest restoration, including its impact on plant responses to phytopathogens, promotion of nutrient uptake, facilitation of seedling adaptation, resource exchange through shared hyphal networks, stimulation of native soil microbial communities, and modulation of gene and protein expression to enhance adaptation to adverse environmental conditions.

## 1. Introduction

Recently, manifestations of climate change have increased in frequency and intensity [1]. These changes have promoted forest fires, water deficits, prolonged droughts, and extreme weather events, which are now common manifestations of environmental degradation [2]. Global warming also threatens the conservation of native forests and their associated biodiversity as they have to cope with harsh environmental conditions [3]. In addition, productive activities such as agriculture and forestry negatively affect the conservation of native forests, mainly through intensive chemical management of soils [4]. As a result, metal(loid)s, xenobiotics, and fertilizers are increasingly reaching native forest soils [5]. As a direct result of environmental and anthropogenic pressures, the extinction rate of plant species and their associated biota is currently increasing [6].

Abiotic stress damages native forest regeneration [7]. In forests, multiple abiotic stresses significantly affect ecosystem services, as these factors also alter soil and forest-associated microbial diversity. In the field of plant–microbe interactions, symbiosis has often been used to describe mutualistic beneficial associations. Root nodule symbiosis and arbuscular mycorrhizal symbiosis are the two most extensively studied symbiotic relationships [8,9]. These interactions are intimate, with at least one partner being obligately dependent on the association as part of its life history [10]. However, we will use the term “symbiosis“ in its literal sense of “living together,” regardless of whether the outcome is beneficial, neutral, or detrimental between the two or more biological species involved [11]. The plant microbiota includes all microorganisms living in and on the plant (bacteria, archaea, fungi, protozoa, and algae) [12]. Part of this microbiota includes plant growth-promoting microorganisms, which have a beneficial effect on plant growth and development. These microorganisms employ direct and indirect mechanisms of plant growth promotion that act simultaneously [13].

The seed is a fundamental structure of plants that contains the genetic information to sustain growth and reproduction, ensuring the continuity of plant species and the development of new generations. Microorganisms can reside on and within the seed and play a critical role in supporting plant health during the early stages of growth. The seed microbiota benefits the seedling in essential processes such as nutrient acquisition, redox homeostasis, modulation of plant secondary metabolism, protection against pathogens, growth promotion, antioxidant activity, and hormone production and modulation [13,14]. The seed microbiota is critical for early plant growth and development. However, it is highly susceptible to changes in forest abiotic conditions, highlighting the importance of vital beneficial symbioses that are critical to the life cycle of forest plants [15]. These microorganisms can positively influence plant responses to environmental stress, suggesting that microbiota can maintain plant adaptation under stress.

Although there are numerous studies that explore beneficial microorganisms at early plant developmental stages, the vast majority associate with annual crops and less than 5% study native forest species. We conducted a systematic review to collect all available scientific information on the juvenile association of forest plants at early stages of development (seed or seedling) in the Scopus database using the keywords “microorganism,” “crop,” and “seed” or “seedling,” and 2530 articles were found. However, if we use the keywords “microorganism,” “native,” “tree,” and “seed” or “seedling,” 91 articles were found, which is 3.6% of the total articles in fruit or crop. After a critical review of the results, 13 articles from 2015 were related to specific genera of beneficial microorganisms. In this review, we summarize the most important studies on plant–microbiota interactions in juvenile native forest trees. We highlight the role of beneficial symbiotic microorganisms in promoting abiotic stress tolerance under climate change, especially at the seedling stage where resistance to challenging field conditions is critical for successful establishment.

## 2. Juvenile Plant–Microbe Interactions

The symbiotic relationship between plants and microorganisms persists throughout the plant life cycle. However, the initial beneficial interactions play a critical role in promoting or hindering ecosystem adaptation rates [16]. Most of our knowledge about beneficial microorganisms during the early stages of plant development comes from studies conducted on crops [17]. However, in recent years, there has been an increasing focus on studying juvenile interactions between forest plants and microorganisms, driven by a growing awareness of the importance of preserving pristine forests [18]. Identifying and managing juvenile plant–microbe interactions is particularly important for sustainable forestry practices, as it provides an alternative for improving plant growth and adaptation rates.

### 2.1. Seed-Associated Microorganisms

Seed-associated microorganisms are part of the diverse pool of beneficial microorganisms that interact directly with the vital organs of plants. This symbiotic relationship includes beneficial microorganisms that are intimately associated with seed structures. Typically, these microorganisms are transferred directly from the parent plant to its progeny through vertical transmission [19]. Other microorganisms are acquired from the surrounding ecosystem through horizontal transmission [20]. Both processes enrich seeds with microorganisms, some of which provide beneficial functions for plant growth. These are called seed endophytes or seed-associated microorganisms [21].

The vertical transmission of some fungal endophytes of grasses, such as *Epichloë*, via seeds has been extensively studied [22]. In many vertically transmitted symbioses, the symbiont is obligate and spends its entire life cycle inside the host, unable to survive in the environment [23]. The leaf-nodulating nitrogen (N) -fixing *Burkholderia* symbionts appear to be obligate endophytic bacteria that have been vertically transferred to the genera *Ardisia*, *Pavetta*, *Psychotria*, and *Sericanthe* [24]. However, there is no evidence for co-speciation between hosts and *Burkholderia*, as the source of the bacteria could be the environment, the parent plant, or both [25]. The presence of bacteria in seeds does not necessarily mean that they originated from the parent, and not all seed-inhabiting bacteria will necessarily colonize seedlings [26].

While the classification of seed endophytes has traditionally been based on the isolation of strains after superficial seed disinfection, we prefer to define seed-associated microorganisms, including surface and endophytic microorganisms. Microbial species transferred to plants can also adopt an endophytic lifestyle and colonize areas outside plant organs, such as the rhizosphere or phyllosphere [27]. Most knowledge about seed-associated microorganisms comes from studies of bacteria interacting with annual herbaceous and agricultural plants [28]. Seed endophytic microbiota are genotype specific [29]. Pseudomonadota, Bacillota, Actinomycetota, and Bacteroidota are phyla that represent some of the most common seed-associated bacteria. Genera such as *Bacillus*, *Acinetobacter*, *Pantoea*, *Enterobacter*, *Paenibacillus*, and *Pseudomonas* are commonly found in seeds [14]. Some of them are beneficial bacteria that promote plant growth directly (biofertilizing and phytostimulating activities) and indirectly (antagonistic activities) [19]. The direct mechanisms allow for an increase in the availability and uptake of nutrients by the plant (biofertilizer activity) and the production and release of secondary metabolites (phytostimulant activity) [30]. An indirect mechanism is biocontrol, where the beneficial microorganisms have antagonistic activity against some plant pathogens [31].

There are two main routes by which endophytic bacteria enter seeds: through the flowers or through the internal pathway (xylem vessels or meristems) [13]. These bacteria have been found in different seed compartments, such as the seed coat, embryo, and endosperm [32]. Endophytic seed-associated bacteria isolated from *Sophora davidii* were vertically transmitted to the next generation of plants [33]. The plant growth promotion induced by seed-associated bacteria is particularly active under adverse growth conditions, primarily by improving tolerance to abiotic stresses such as metal(loid)s or water deficit, and by enhancing the production of hydrolytic enzymes [34].

Fungi and yeasts are also commonly reported as seed-associated microorganisms. The genera *Alternaria*, *Aureobasidium*, *Cladosporium*, *Epicoccum*, *Phaeosphaeria*, *Phoma*, *Pyrenophora*, *Stagonospora*, *Chaetomium*, *Fusarium*, *Microdochium*, *Stemphylium*, and *Xylaria* have been described as major taxa in seeds [35]. A study by Yang, et al. [36] demonstrated differences in the composition of leaf, root, and soil mycorrhizae in 13 different tree species, suggesting that fungal mycorrhizae do not systematically infect the species tested. Similarly, *Carya illinoinensis* may benefit from interactions with fungal endophytes such as *Beauveria bassiana*, which play a positive role in the management of the insect pests *Melanocallis caryaefoliae* and *Monellia caryella* [37].

The global diversity of seed-associated microorganisms in woody plants remains to be studied. Improving nutrient uptake, biotic or abiotic stress tolerance, phytohormone production, and protection against common phytopathogens is of vital importance for woody plants, especially during the early developmental stages when seedlings need to adapt to specific ecosystem characteristics [38]. Studies carried out on *Citrus limon* have shown that the bacterial genera *Cutibacterium* and *Acinetobacter* and the fungal genera *Cladosporium* and *Debaryomyces* were among the most abundant taxa in seeds and shoots, with some bacterial taxa being vertically transferred from the endophytic microbiota to seeds [39]. Studies on the tree *Anadenanthera colubrina* showed that *Methylobacterium* and *Staphylococcus* spp. were the main beneficial strains associated with the seeds. At the same time, *Friedmaniella*, *Bifidobacterium*, *Delftia*, *Anaerococcus*, and *Actinomyces* were reported as novel seed-associated taxa [40]. Management of seed-associated microorganisms is a strategy that can be implemented to enhance forest plant growth and establishment rates. However, critical issues such as pathogenicity and compatibility need to be addressed in laboratory trials before the widespread management of seed-associated microorganisms can be implemented for forest restoration.

### 2.2. Beneficial Microorganisms at the Plantlet Stage

Under wild conditions, plants stimulate the growth of specific microorganisms, allowing for the transmission of soil-borne microorganisms [41]. Beneficial microorganisms such as mycorrhizal fungi, endophytic fungi and bacteria, and rhizosphere-inhabiting taxa can influence plant performance and contribute positively to natural forest regeneration. Soils affected by environmental stress often suffer severe changes in the diversity of active soil microorganisms, altering the microbial diversity potentially associated with plant roots [42]. In this context, plants harboring endophytic microorganisms or those that establish non-specific interactions with microorganisms inhabiting the soil substrate have a higher probability of successful establishment [43,44]. Therefore, microbial symbioses play an essential role in the establishment of native plants in the ecosystem by providing nutritional and metabolic benefits to the plants [45].

Plant nurseries play a critical role in ecosystem restoration by promoting the reforestation of degraded areas with native plants, producing numerous individuals that can be directly transplanted into the ecosystem [46]. However, these plants are typically grown under optimal nutritional, hydrological, and climatic conditions. These conditions differ from the harsh environments of degraded ecosystems where plants face challenges such as water deficit, nutrient-poor soils, and biotic stresses [47]. The inoculation of seedlings with beneficial microorganisms, either as single strains or consortia, has been shown to have a positive effect on the performance of forest trees under both nursery and field conditions [48]. Pre-transplant inoculation with plant growth-promoting rhizobacteria can enhance seedling growth primarily through their ability to produce phytohormones, biocontrol phytopathogens, fix N, exhibit 1-amino-cyclopropane carboxylic acid (ACC) deaminase activity, and induce systemic resistance [49]. However, inoculation with root-associated fungi, including mycorrhizae and other beneficial endophytic taxa, can increase the water and nutrient uptake surface by exploring soil colloids and dissolving insoluble phosphate compounds, thereby extending water availability beyond the root/rhizosphere interface [50]. These resources are exchanged with the plant in exchange for carbon (C) and other plant-derived metabolites [51]. Even in invasive plant species, studies have demonstrated the direct interaction of juvenile organs with beneficial microorganisms that can influence growth and development through interactions with beneficial genera such as *Rhizophagus* and *Bacillus*, which are directly involved in nutrient solubilization and biocontrol of phytopathogens, respectively [52]. In addition, the induction of metabolic and genomic changes in symbiotic plants is one of the primary mechanisms activated by microorganisms to cope with environmental stress [53].

## 3. Metabolic Responses of Plants and Their Associated Microorganisms to Abiotic Stress

Plants can regulate seed endophytes in response to environmental stressors and transfer them to the next generations. They use these microorganisms during their establishment phase and throughout their entire lifecycle. Several climatic variables trigger this response and serve to recruit microorganisms from the environment that help to adapt to stressful conditions [54]. In addition, the genes responsible for hormone production in plant growth-promoting rhizobacteria are influenced by several stress factors affecting the soil and rhizosphere. These factors include acidic pH and osmotic stress, and root exudates that also influence their regulation [55]. Vertically or horizontally transferred microorganisms can enhance plant adaptation to adverse environmental conditions.

Beneficial microorganisms are crucial to mitigate the current impacts of climate change on the regeneration of native forests. Among them, the beneficial effects of symbiotic interactions during the juvenile stage of plants are essential for promoting growth under the severe environmental conditions resulting from climate change. However, analyzing the responses of forest plants to individual abiotic stresses is often challenging since the negative consequences under current climate change conditions are the accumulation of multiple abiotic stresses. This is where microbial associations play a critical role in supporting plant growth and establishment. For example, endophytic *Bacillus* spp. have been identified as potent multi-stress-tolerant taxa with beneficial plant growth-promoting traits [56]. In addition, they exhibit positive effects against phytopathogens through secondary metabolite synthesis, biofilm formation, and quorum sensing to modulate plant metabolism [57]. Many other examples highlight the role of fungal associations, such as mycorrhizal fungi, in adapting to multiple stresses, primarily by enhancing growth and inducing gene expression that activates stress-responsive genes [58].

### 3.1. Water Deficit

Drought can have a significant negative impact on plant growth, primarily through the inhibition of gas exchange. The main plant response to water shortage is stomatal closure, which reduces water loss through transpiration. This process inevitably leads to a decrease in C assimilation and, subsequently, a decrease in overall biomass production [59]. This immediate response also contributes to a decrease in the mass flux of water-soluble nutrients from the soil, resulting in reduced nutrient uptake and utilization, exacerbating the negative effects of drought [60]. In contrast, when plants experience prolonged water stress, stomatal closure is typically accompanied by metabolic constraints that result in a downregulation of photosynthetic rates due to the limitation of ribulose-1,5-bisphosphate synthesis [61]. Recently, the modes of action by which soil microorganisms enhance plant water conservation and recovery mechanisms have been succinctly summarized, highlighting soil microorganisms as potential resources for the development of biological strategies to support plant growth [62,63]. Consequently, microorganisms directly influence the response of plants to water deficits, whether such deficits are of short or prolonged duration.

Root-associated microorganisms can enhance plant performance under water deficit by synthesizing osmoprotectants and other compatible solutes. These compounds play a critical role in maintaining intracellular water balance and protecting against dehydration by stabilizing proteins and cellular structures [64,65]. Endophytic bacteria can also increase abscisic acid (ABA) levels, influencing plant metabolism to better adapt to the stressful conditions caused by a water deficit [66]. Furthermore, the establishment of microbial symbioses under water limitation enhances the expression of stress-related genes that typically improve plant performance [67]. Similarly, changes in the production of antioxidant enzymes such as superoxide dismutase (SOD), catalase (CAT), and peroxidase help to scavenge reactive species [68]. Beneficial root-associated fungi can also increase plant water availability by expanding the root absorption surface [69]. Similarly, microorganisms can optimize water use directly or indirectly by regulating stomatal opening and closing [70].

### 3.2. Salinity

Although salt and water stress may appear to be related in some ways, it is beneficial to study them separately to gain a full understanding of how they affect forest species and how they can be effectively managed in different contexts. This will provide better knowledge for the management of forest species under different environmental conditions. Salt stress is one of the most complex types of stress because it involves an effect on water and therefore the osmotic state of the plant, oxidative stress, and ionic toxicity accompanied by nutrient imbalance. It is a harmful condition that affects seed germination and seedling growth, mainly through a continuous decrease in water uptake and disruption of essential enzymatic activities. It causes an imbalance in hormone crosstalk, antioxidant activities, ionic homeostasis, and reactive oxygen species (ROS) that disrupt essential biomolecules such as DNA and proteins [71]. Plant growth is usually reduced by high concentrations of salts, which can induce stomatal closure and, thus, a reduction in carbon dioxide assimilation, which reduces plant metabolism [72]. In the soil, salinity also leads to severe changes in microbial diversity, affecting the diversity of beneficial microorganisms with potential roles in promoting plant growth [73].

Soil microorganisms are affected by high concentrations of salts in the soil solution. In microorganisms sensible to salinity, some essential genes involved in central metabolism are repressed, and proteins related to elongation factors, chaperone, and cell multiplication are negatively affected in the presence of high concentrations of salts [74]. Salinity reduces the rhizobial colonization of the rhizosphere by inhibiting the synthesis of bacterial surface molecules such as glucans, lipopolysaccharides, and exopolysaccharides, which are essential for their interaction with the plant [75]. However, this stress condition stimulates the development of halotolerant strains with a potential role in promoting plant growth [76].

Symbiotic microorganisms, such as arbuscular mycorrhizal fungi, selectively enhance nutrient uptake, including potassium (K) uptake, while reducing sodium uptake [77]. In addition, these microorganisms enhance plant resistance to salinity through mechanisms such as protein expression, increased water and nutrient uptake, and defense against phytopathogens. Recent evidence also suggests that mycorrhizal fungi are effective candidates for mitigating salt stress in plants [78]. Mechanisms that reduce ROS accumulation are among the mechanisms by which beneficial microorganisms help to scavenge ROS and protect plant tissues from salinity. Seed-associated microorganisms can also improve soil health, as plants carrying halophytic strains can improve soil structure, nutrient cycling, and organic matter decomposition, ultimately improving the water-holding capacity and overall soil nutrient status. Similarly, an inoculation of *Zelkova serrata* with *F. mosseae* increases the photosynthetic rate and leaf P, K, and magnesium (Mg) content under salinity stress conditions [79].

### 3.3. Heat Stress

High temperature and direct ultraviolet radiation directly affect plant performance under heat stress, affecting seed germination and plantlet establishment. Among abiotic stresses, extreme and rapid temperature variation is one of the most detrimental factors affecting forest plant growth [80]. Temperature is critical for promoting seed germination and affects water movement in plants under heat stress. Similarly, soil water content and water availability to plants in soils exposed to high temperatures constantly decrease due to heat-induced water evapotranspiration [81]. Plants have evolved anatomical and physiological modifications to survive under heat stress, mainly by producing compatible solutes that induce molecular changes, leading to an osmotic adjustment and restoration of redox homeostasis [82,83].

Beneficial microorganisms, such as *Pseudomonas* and *Trichoderma*, can enhance the expression of heat shock proteins (HSPs), which help maintain protein integrity [84,85]. Microorganisms that produce HSPs can interact directly with plant organs, from seeds and seedlings to mature plants. An improved nutrient availability and water balance, resulting from an increased root growth or improved absorption surface, are among the beneficial effects of microorganisms that indirectly affect plant performance by enhancing growth and overall metabolic activity in response to heat stress [86]. However, the microbially induced modulation of plant hormones such as ABA and jasmonate can trigger metabolic responses that alter the physiological state of the plant to enhance stress tolerance [87]. The microbial priming of plant growth-promoting microorganisms, which refers to the enhanced response of an organism to a second stress event after a previous temporally limited minor stress, can prepare beneficial microorganisms for the detrimental effects of prolonged heat periods [88]. Heat-tolerant strains of *Bacillus safensis* mitigate the deleterious effects of heat stress in inoculated plants through increased levels of ACC deaminase, indole-3-acetic acid (IAA), gibberellic acid, and the production of kinetin and exopolysaccharides [89]. Soil fungi such as *Trichoderma koningii* increase plant tolerance primarily through an enhanced production of antioxidants [90]. Arbuscular mycorrhizal fungi also improve heat stress tolerance in inoculated plants by increasing the expression of genes involved in heat stress tolerance [91]. Endophytic fungi, such as *Paecilomyces formosus* and *Penicillium funiculosum*, can also improve the physiological responses of plants under high temperature and drought stress [92].

### 3.4. Cold Stress

Low temperatures severely affect plant metabolism, compromising plant growth and survival. In the early stages of plant development, low temperatures severely reduce seed germination and plantlet establishment [93]. Reduced leaf expansion and chlorosis are often reported in plants growing under cold stress, indirectly affecting photosynthesis and altering the activity of essential enzymes [94]. The adverse effects on plant organs can ultimately induce necrosis, threatening the functionality of essential plant organs [95]. The stability of membranes is also affected, which is a significant adverse effect [96]. However, cold stress-adapted plants can induce the expression of specific fatty acids that alleviate cold stress at the cellular level [97].

Psychrophilic microorganisms enhance plant tolerance to cold stress by producing antifreeze proteins, cryoprotectants, and osmoprotectants to prevent cellular damage caused by low temperatures [98]. Microorganisms can also synthesize phytohormones that enhance the physiological responses of plants under cold stress [99]. Metabolic pathways involved in low-temperature resistance are also stimulated by the action of microorganisms [100]. Psychrophilic microorganisms associated with plants can also maintain vital plant functions, including nutrient solubilization, phytohormone production, and biocontrol at low temperatures [101]. *Bacillus* spp. isolated from the rhizosphere of plants growing on the Qinghai–Tibetan Plateau demonstrated the ability to be cold-adapted and promote the growth of *T. aestivum* seedlings under cold conditions [102]. Similarly, psychrotolerant *Pseudomonas* sp. and *Curtobacterium* sp. can promote growth under cold stress when applied as a consortium [103]. A bacterial consortium consisting of *Bacillus cereus*, *B. subtilis*, and *Serratia* sp. attenuates cold stress-induced injury by activating specific transcription factors [104]. Psychrophilic *Bacillus* spp. alleviate cold stress mainly through the expression of genes involved in phytohormone metabolism [105]. Mycorrhizal fungi also improve plant growth under cold stress, mainly by activating antioxidant defense mechanisms, accumulating protective molecules, improving growth, photosynthesis, osmotic potential, and reducing membrane damage [106]. Similarly, fungal endophytes such as *Penicillium rubens* and *Penicillium bialowienzense* can modify gene expression in inoculated *Vaccinium corymbosum*, resulting in higher photochemical efficiency and less oxidative stress compared to uninoculated plants [107].

### 3.5. Mineral Deficiency

In forest ecosystems, nutrient deficiency is the major limiting factor for sustaining plant growth. In plants, the gene expression of membrane transporters involved in nutrient uptake is often upregulated under nutrient limitation [108]. Similarly, an increased exudation of metabolites such as carboxylates, protons, sugars, amino acids, and proteins is characteristic of plants that must overcome mineral deficiencies. Such exudation can directly affect nutrient availability in the soil solution or stimulate microbial symbioses with soil-borne microorganisms.

Microorganisms can produce organic acids that contribute to mineral solubilization. Stimulating microbial communities that can support growth in the early stages of plant development is essential under nutrient deficiencies. Symbiotic microorganisms such as arbuscular mycorrhizae and N-fixing bacteria are helpful in nutrient mobilization. Extraradical hyphae of root-associated fungi can explore the bulk soil for nutrient resources [109]. Soil microorganisms indirectly enhance nutrient uptake by improving root growth and nutrient uptake surfaces [110]. One of these mechanisms is related to the synthesis of auxins and cytokinins by root-associated microorganisms [111]. Seed-associated microorganisms such as *Bacillus* sp., *Citrobacter* sp., *Flavobacterium* sp., and *Pantoea* sp. can enhance nutrient solubilization [112]. Ribeiro et al. [113] determined the ability of endophytic *Bacillus* spp. to improve the N, phosphorus (P), and K content of inoculated plants. Endophytic strains of *Aspergillus terreus*, *Lecanicillium* sp. *Pseudomonas bijieensis*, and *Priestia megaterium* have also been shown to induce improved Zinc concentration and NPK content in inoculated plants [114]. Fungal endophyte inoculation also helps to improve the growth and grain yield of plants growing under nutrient starvation conditions [115].

### 3.6. Metal(loid)s

Although metal(loid)s are typical components of soil and plants, and these elements are necessary cofactors for many enzymes, metal(loid)s in excess negatively affect plant metabolism. Specifically, one of the most important negative effects of metal(loid)s is their effect on protein synthesis, structure, and the corresponding function of proteins [116]. In addition, metal(loid)s can replace cofactors in many metabolic reactions, leading to inefficient metabolic processes such as photosynthesis, membrane damage, electrolyte imbalance, reduced growth, and reduced root hairs. These effects ultimately lead to reduced water uptake, nutrient imbalance, and increased DNA damage [117].

Symbiotic fungi and bacteria have evolved adaptive mechanisms to avoid harmful damage to their internal metabolism, including the production of siderophores, organic acids, and exopolysaccharides [118]. Symbiotic fungi can produce metabolites that can sequester metalloids in soil solution, thus preventing uptake by plants [119]. Similarly, they can sequester the metal(loid)s directly within fungal structures [120]. Bacteria also employ mechanisms to prevent plant uptake, such as colonization and biofilm formation on root surface [121]. A core microbiome is transmitted to the next generations of *Noccaea caerulescens* seeds produced in metal-rich soils [122]. This core microbiome prepares plants to better cope with the harsh conditions of polluted soils by inducing metabolic and physiological changes that favor metal accumulation in plants without compromising essential metabolism. Arbuscular mycorrhizal fungi have also demonstrated a role in promoting tolerance by alleviating metal(loid)s toxicity in roots [123]. Hachani et al. [124] demonstrated that ectomycorrhizal fungi enhanced the establishment of *Pinus halepensis* seedlings in soil contaminated with multiple heavy metals (Pb, Zn, and Cadmiun).

## 4. Role of Juvenile Plant–Microbe Symbiosis in Native Forest Regeneration

**Figure 1:**
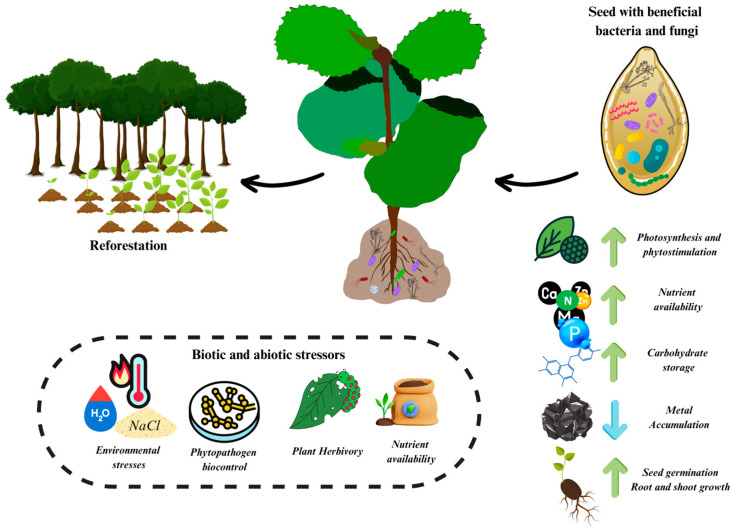
Schematic representation of the integration of beneficial plant-growth promoting microorganisms at the juvenile plant stage of native tree species, whether at the seed or plantlet stage.

Considering the beneficial effect of fungi and bacteria on plant growth promotion, the identification of beneficial symbiotic relationships between microorganisms and seedlings is essential to promote plant adaptation after successful seed germination. However, in forest plants, most microbial symbioses (except mycorrhizae and rhizobia) during the juvenile stage are largely unknown (Table 1). Therefore, understanding the interactions between different forest plants and soil microorganisms is crucial for the comprehensive management of microbial symbioses to improve the quality of plants produced in nurseries for reforestation. In this context, it is essential to know the diversity of beneficial bacteria and fungi that interact with forest plant seedlings. These microorganisms can be integrated into the production process of native plant nurseries. This integration ensures that seeds and plantlets receive beneficial microorganisms, ultimately improving the performance of these plants in the field (Figure 1).

The incorporation of beneficial microorganisms is crucial for generating robust plantlets for reforestation programs. This approach aims to counteract negative consequences such as environmental stresses, phytopathogen-related diseases, herbivore pressures, and limited nutrient availability. By inoculating these microorganisms, it is expected that multiple benefits will be realized, including increased seed germination, diminished metal accumulation, improved carbon storage, enhanced nutrient availability, and overall improvements in photosynthesis and phytostimulation. This comprehensive strategy supports the health and vitality of native tree species, thus contributing significantly to the success of reforestation efforts.

### 4.1. Promoting the Adaptation of New Plantlets

Seed germination and seedling establishment are critical processes in the life cycle of forest plants. Many forest understory plants face a challenging environment characterized by competition, low light availability, predation, and the adverse effects of climate change. In this context, beneficial symbioses enhance plant growth and overall metabolism. Juvenile plant–microbe interactions can be a powerful tool to enhance seedling robustness during field establishment. Therefore, the inoculation of seedlings with beneficial plant growth-promoting microorganisms represents an alternative to chemical fertilization and phytopathogen control. However, it is important to consider the compatibility between specific soil microorganisms and plant species.

Previous studies investigated the potential of *Bacillus* sp. to improve the growth of *Populus euramericana* by increasing aerial biomass and photosynthetic rate [125]. In addition, *Bacillus* sp. and *Paenibacillus* sp. strains were used to improve the growth of *Abies nordmanniana.* Specifically, *Bacillus* sp. improved seed germination, storage carbohydrate levels and induced systemic resistance by enhancing the activities of glutathione reductase and glutathione S-transferase during the juvenile developmental stage. On the other hand, *Paenibacillus* sp. increased root growth, shoot soluble carbohydrate content, starch content, and chlorophyll content. Therefore, the co-inoculation of both strains was suggested to improve growth under greenhouse and field conditions [126]. Similarly, the inoculation of *Pseudomonas* sp. increases the germination of *Santalum album* as well as the growth of *Pongamia pinnata* and *Araucaria angustifolia* through the production of ammonium, IAA, siderophores, and phosphate solubilization [127,128,129]. Similarly, inoculation of the seed endophytes *Methylobacterium* sp. and *Kineococcus* endophyticus on *Populus deltoides* improved plant growth in seedlings and reduced metal accumulation [130].

The mycorrhizal fungus *Funneliformis mosseae* induces resistance to metal stress, such as cadmium and lead, in *Cupressus arizonica*, and *Robinia pseudoacacia* by reducing electrolyte leakage and increasing translocation capacity in roots and stems [131,132]. Under water stress, inoculation with *Rhizophagus* sp. reduces oxidative damage in *C. arizonica* and *Cupressus atlantica* by increasing enzymatic antioxidants with CAT and SOD [133,134]. Similarly, the inoculation of *Quercus brantti* with *Microbacterium* sp. and *Streptomyces* sp. under water stress increased the phosphate solubilization [135]. Azizi et al. [136] demonstrated that inoculation of *Myrtus communis* with fungi and bacteria induced drought resistance by reducing electrolyte leakage, malondialdehyde and proline content, and mitigating oxidative pigment loss under drought conditions through positive regulation of antioxidant defenses. Similarly, Hashem et al. [137] showed that fungal and bacterial inoculation induced acquired systemic resistance in *Acacia gerrardii* under salt stress conditions by increasing the content of total lipids, phenolics, and fiber, as well as the content of osmoprotectants such as glycine, betaine, and proline.

### 4.2. Protecting the Forest Trees against New Phytopathogens

Climate change also alters the symbiotic lifestyle of some endophytes, which, depending on the physiological state of the plant, may become opportunistic pathogens and cause diseases that affect plant health. For example, several pathogens have recently been described in the ancient Andean monkey tree *Araucaria araucana*, including *Diplodia mutila* and *Pewenomyces kutranfy* [138,139]. However, symbiotic interactions of plants play a critical role in promoting the resistance to phytopathogens. Symbiotic fungi and bacteria, such as *Bacillus*, *Paenibacillus*, and *Pantoea* have been isolated from wild plants and have demonstrated biocontrol potential against pathogenic organisms [140]. Similarly, the yeast *Aureobasidium pullulans* has been classified as an effective antagonist against major foliar pathogens [141]. This yeast has been isolated from wild environments, such as deserts and tree leaves [142,143]. The ability of this yeast to grow in cold conditions can effectively manage seed storage in native forest seed banks, as some psychrophilic opportunistic pathogens can also affect seed viability [144]. Similarly, the genus *Bacillus* sp. has demonstrated great biocontrol capacity against pathogens of *Camellia oleifera* and *Juglans regia* through the production of enzymes such as chitinase, in addition to promoting growth through phytohormones such as auxins and phosphate solubilization [145,146]. Similarly, inoculation of *Anacardium othonianum* with *Acinetobacter lwoffi* and *Pantoea agglomerans* increase the growth promotion and biocontrol against *Fusarium oxyspotum* [147].

**Table 1:** Principal studies analyzing the growth promotion of beneficial bacteria and fungi in native forest tree plantlets.

Microorganisms	Plant Species	Mechanisms	References
*Bacillus subtilis*	*Populus euramericana* *Populus deltoides × Populus nigra*	Enhanced seedling height by 62% and total biomass by 37% after 120 days. The photosynthetic rate increased by 54%.	[125]
*Rhizophagus manihotis* *Rhizophagus Agregatus* *Rhizophagus fasciculatus* *Acaulospora* sp.	*Cupressus atlantica*	Increased the relative water content and water potential under water deficit stress. Increase contents of proline and of soluble sugars. Increase Superoxide dismutase (SOD) and catalase (CAT) activities.	[133]
*Funneliformis mosseae* *Diversispora tortuosa*	*Gleditsia sinensis*	Increase seedling height, basal diameter, dry biomass. Increase chlorophyll concentrations and photosynthetic rates. Increased phosphorus (P) and potassium (K) content in leaf, stem, and root, and increased nitrogen (N) content in the leaf and stem.	[148]
*Bacillus subtilis* *Claroideoglomus etunicatum* *Rhizophagus intraradices* *Funneliformis mosseae*	*Acacia gerrardii*	Induce acquired systemic resistance against adverse impact of salt stress. Improvement in the nutritional value in terms of increase in total lipids, phenols, and fiber content. Increased content of osmoprotectants such as glycine, betaine, and proline.	[137]
*Rhizophagus intraradices* *Funneliformis mosseae* *Pseudomonas fluorescens*	*Cupressus arizonica*	Induce resistance under Cadmium (Cd) stress condition. Increase P, K and iron concentrations, height, shoot dry weight, proline content and reduced electrolyte leakage percentage.	[131]
*Bacillus licheniformis*	*Camellia oleifera*	Production lytic enzymes chitinase and β-1,3-glucanase that can inhibit foliar pathogens by 37.4% *(Botrytis cinerea*) to 50.5% (*Pestalotiopsis karstenii*). Increased the total N and P contents in the soils. Increased root dry weight and production the phytohormone auxin.	[145]
*Bacillus velezensis*	*Juglans regia*	Production lytic enzymes chitinase, protease, and β-l,3-glucanase activity and degraded the cell wall of *Colletotrichum gloeosporioides*. Production indole-3-acetic acid (IAA) and exhibited the potential for ammonium production and phosphate solubilization.	[146]
*Pseudomonas fluorescens*	*Santalum album*	Biopriming at 100% for 8 days recorded the highest germination percentage (88%).	[127]
*Pseudomonas aeruginosa*	*Pongamia pinnata*	Ammonia production, IAA production, siderophore production and was observed to promote solubilization of phosphate, silicate and zinc in the plate assay.	[128]
*Funneliformis mosseae* *Rhizophagus irregularis* *Pseudomonas putida* *Pseudomonas fluorescens*	*Myrtus communis*	Drought resistance, improved leaf physiology, reduced electrolyte leakage, malondialdehyde, and proline concentrations and mitigated oxidative pigment losses under drought through upregulation of the antioxidant defense as evidenced by non-enzymatic antioxidant accumulation.	[136]
*Funneliformis mosseae* *Diversispora tortuosa*	*Zelkova serrata*	Induce resistance salt stress. Increasing the leaf photosynthetic ability and biomass accumulation by reducing sodium content, increasing P, K^+^, and magnesium content, as well as by enhancing photosynthetic pigments content and the stomatal conductance of leaves.	[79]
*Pseudomonas* sp. *Bacillus subtilis* *Bacillus amyloliquefaciens*	*Araucaria angustifolia*	IAA, Siderophores production, inorganic phosphate solubilization.	[129]
*Rhizophagus* *irregularis* *Funneliformis mosseae* *Pseudomonas fluorescens*	*Cupressus arizonica*	Reduction oxidative damage in water stress (reduce hydrogen peroxide and MDA) and increase the enzymatic antioxidants (CAT, SOD, glutathione peroxidase, ascorbate peroxidase).	[134]
*Funneliformis* *mosseae*	*Robinia pseudoacacia*	Induce resistance lead (Pb) stress. Increased the root activity and root tolerance index. Inoculated plants had greater accumulation and translocation capacities for Pb in the roots and stems.	[132]
*Bacillus* spp. *Paenibacillus* spp.	*Abies nordmanniana*	Improved seed germination and produced IAA. Increased plant root growth, especially by inducing secondary root formation, under in greenhouse conditions.	[126]
*Methylobacterium* sp. *Kineococcus endophyticus*	*Populus deltoides x (Populus trichocarpa x Populus maximowiczii)*	IAA production, phosphorus solubilization. reduced the bioaccumulation of Zn and Cd.	[130]
*Pseudomonas frederiksbergensis*	*Populus euramericana*	Phosphate-solubilizing activity, growth rate and organic acid secretion (high concentrations of gluconic, 2-ketogluconic, pyruvic, maleic and malic acids).	[149]
*Trichoderma harzianum* *Trichoderma asperiana*	*Cabralea canjerana* *Cedrela fissilis* *Cordia trichotoma* *Erythrina cristagall* *Luehea divaricata*	Increase the supervival rates and height and diameter of plants.	[150]
*Caballeronia sordidicola*	*Picea glauca x engelmannii*	Help in biological nitrogen fixation in limit soil nitrogen and enhanced seedling length and biomass by nearly	[151]
*Claroideoglomus etunicatum* *Acaulospora* sp. *Rhizobium* sp. *Burkholderia* sp.	*Schizolobium parahyba*	The application of microorganisms increased wood yield by about 20% compared to the application of fertilizer alone.	[152]
*Microbacterium* sp. *Streptomyces* sp.	*Quercus brantii*	The inoculation of the bacteria increased the rate of phosphate solubilization, improving root growth and seedling weight under water stress conditions.	[135]
*Acinetobacter lwoffii* *Pantoea agglomerans*	*Anacardium othonianum*	Auxin production, phosphate solubilization, production of phosphatases, siderophores, and biocontrol against *Fusarium oxysporum.*	[147]
*Caballeronia sordidicola*	*Pinus contorta*	Inoculation of diazotrophic bacteria increased the fixation of 49-50% of the host atmospheric nitrogen, and increased seedling length and biomass up to 1.5 and 4 times, respectively.	[153]
*Rhizophagus clarus* *Gigaspora margarita*	*Chizolobium parahyba*	In the absence of P, growth variables (height, dry matter area, root dry matter, leaf area, stem diameter) increased in relation to control plants. N, P, Ca and Mg contents were also influenced by fungal inoculation.	[154]

### 4.3. Promoting Nutrient Mineralization

Plant growth-promoting microorganisms, which are active in the early stages of the life cycle of forest plants, are critical in promoting plant growth by providing the essential nutrients needed for germination. Nutrients such as C, N, and P can be directly supplied by root-associated microorganisms that possess the enzymes necessary to dissolve these nutrients from the environment. The production of organic acids is one of the mechanisms by which symbiotic microorganisms enhance P solubilization, thereby improving the growth of *P. euramericana* [149]. However, the continuous exudation of organic acids has multiple effects on the soil by activating rhizosphere-inhabiting microorganisms that enhance nutrient mineralization and abiotic stress tolerance [155]. Wang et al. [148] showed that inoculation of *F. mosseae* in *Gleditsia sinensis* increased leaf N, P, and K contents, chlorophyll concentrations, and photosynthetic rates. Brito et al. [154] showed that the inoculation *Chizolobium parahyba* with *Rhizophagus clarus* and *Gigaspora margarita* in absence of P, increase N, P, Ca, and Mg. Similarly, the diazotropic bacteria *Caballeronia sordidicola* increase the N fixation in *Picea glauca x engelmannii* and *Pinus contorta* in limit soil nitrogen [151,153]. The inoculation of forest seeds or seedlings with microorganisms directly involved in essential nutrient mineralization and with multi-stress resistance is an alternative approach that can improve the establishment rates of forest plants in the ecosystem.

### 4.4. Moving Nutrients and Signals through Shared Hyphal Networks

The microbial symbiosis between seedlings and root-associated fungi begins in the early stages of plant development. If fungi are not initially part of the seed-associated microbiome, they can be stimulated from the rhizosphere where seeds begin to produce plant metabolites that stimulate spore germination and the growth of beneficial fungi, such as arbuscular mycorrhizal fungi. As a result, a complex hyphal network is formed in the soil that connects plant roots through common fungal hyphae. These hyphal networks have effectively transported resources or chemical signals between neighboring plants [156]. The establishment of hyphal networks involving both mycorrhizal fungi and rhizospheric soil fungi improves soil health by exploring the bulk soil and contributing to specific deposition of organic C stocks, nutrient solubilization, improved stability, and water storage in forest soils [157]. Therefore, the provision of compatible root-associated taxa is essential to improve the establishment success of forest tree seedlings in the field. Griebeler et al. [150] and Cely et al. [152] demonstrated the inoculation with beneficial microorganism in seedling increase the survival rates and growth promotion in exclusive applications and in combination with fertilizer.

### 4.5. Stimulating Native Soil Microbial Communities

A plant growing in soil can stimulate dormant microorganisms that react in response to the metabolic influence of root exudates [158]. The production of low-molecular weight organic acids, pH changes, sugars, and amino acids excreted into extraradical media are detected by bacteria, some of which increase their metabolism and change the relative abundance of specific taxa in the rhizosphere. This stimulation is strongly dependent on the fingerprint of root exudates, which varies according to plant species or even the specific metabolic state of the individual plant [159]. These root-associated microbial communities are sometimes necessary for the life cycle of forest plants, influencing critical life stages such as seed germination and plantlet growth, and establishing specific microbial interactions that are essential for the maintenance of soil ecosystem services associated with microorganisms [160].

### 4.6. Change in the Expression of Genes and Proteins Involved in Plant Adaptation

The adaptation of forest plants in pristine ecosystems is a complex process where, in addition to the adverse effects of climate change, seeds must find the optimal temperature and water availability for successful germination. In addition, phytopathogens, insects, nematodes, and herbivores are the major constraints to plant growth in nature. Mycorrhizal fungi and microbial endophytes have been shown to positively regulate the expression of specific genes that modulate the response of symbiotic plants to abiotic and biotic stresses that can affect plant growth at early stages of development. Such changes in gene and protein expression often result in improved plant growth and establishment rates in challenging ecosystems [161]. Due to the limited knowledge of microbial endophytes in native forest trees, the specific effects of beneficial microorganisms need to be addressed to determine the mechanisms promoted by inoculation and their potential use in forest restoration.

## 5. Concluding Remarks

Global knowledge about microbial interactions of forest trees is mainly focused on the advanced stages of plant development. In contrast, the diversity of active microorganisms at the seed–seedling stage remains largely unexplored. Improving the establishment of forest plants in the field is a global priority, as many pristine forests worldwide are facing environmental pressures triggered by rapid climate change, minimizing the vital roles that forests play in C storage. Particularly important are plants experiencing a continuous decline in their population numbers and generating nonviable seeds, posing a severe threat to the successful establishment of the next generation. Providing plantlets with beneficial microorganisms from the seed or plantlet stage is necessary to improve the performance of plants used in reforestation programs or cultivated in nurseries for native forest reforestation. This review highlights the knowledge gap regarding juvenile microbial interactions of forest plants and summarizes examples of forestry species-beneficial microorganism interactions, which are essential to support plant establishment under the severe scenarios driven by climate change. Further research on juvenile plant–microbe interactions in forest plants must be explored to contribute to maintaining biodiversity and the ecosystem services associated with pristine forests. The compatibility between beneficial microorganisms and specific forest trees should be considered when designing specific reforestation programs.

## Data Availability

Not applicable.
